# Supplementary material for: Affordable RFID loggers for monitoring animal movement, activity, and behaviour
Source: PLoS One. 2022 Oct 27;17(10):e0276388. doi: 10.1371/journal.pone.0276388 (PMC9612574; doi:10.1371/journal.pone.0276388)
Supplement: S1 Table — (DOCX) [file pone.0276388.s001.docx]

**Supplementary S1**

**Table 1.** List of components with source and approximate price for constructing a single unit (as of December 2021 in Australia)

| *Product* | *Quantity* | *Source* | *Price (AUD$)* |
| --- | --- | --- | --- |
| Trovan ISO Microchip All-in-One | 1 | Microchips Australia | $8.90 |
| RFIDLOG animal tag RFID data logger | 1 | Priority 1 Design Pty ltd. | $75.00 |
| RFIDCOIL-160A RFID coil antenna 160mm | 1 | Priority 1 Design Pty ltd. | $6.50 |
| 6 Pin 0.1 Header with Crimp Pins - 2.54 pitch | 1 | Jaycar | $0.75 |
| Turnigy Rechargeable Battery AA 2550mAh NiMH | 6 | Hobby King | $15.06 |
| 6 X AA 2 BY 3 side-by-side Battery Holder | 1 | Jaycar | $2.35 |
| Solder | 1 | Jaycar | $0.80 |
| Plastic container (approx. 450mL) | 1 | Supermarket | $4.60 |
| Silicone gel | 1 | Bunnings | $0.55 |
| 1.5mm Heat shrink Tubing | 1 | Jaycar | $1.45 |
| 7mm Plywood | approx. 40 x 20cm | Bunnings | $7.00 |
| 7mm White Flat Cable Clips | 4 | Bunnings | $2.50 |
| Total |  |  | $125.46 |
