# Supplementary material for: Affordable RFID loggers for monitoring animal movement, activity, and behaviour
Source: PLoS One. 2022 Oct 27;17(10):e0276388. doi: 10.1371/journal.pone.0276388 (PMC9612574; doi:10.1371/journal.pone.0276388)
Supplement: S1 File — (PDF) [file pone.0276388.s002.pdf]

Instructions for building RFID readers and housing.

As described in *Harrison and Kelly (2022).*

The main component of the RFID logger is the printed circuit board (PCB; Fig. 1). Before you start soldering, ensure that you are wearing rubber soled shoes, and have removed any items from your work area that could conduct substantial static charge (plastic items, excess electrical items such as phones etc) to avoid accidental surges to the PCB.

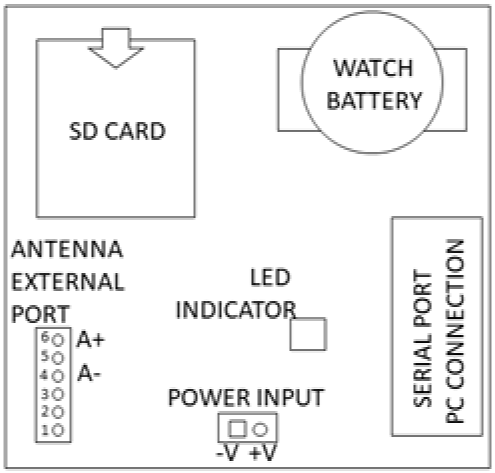

Figure 1. *Schematic drawing of Priority 1 Designs (AU) RFIDlog printed circuit board.*

1. Attaching the external power source (battery pack)

The PCB has a space for a small CR2032 3V lithium battery. This battery only powers the internal clock (which keeps the time and date constant across uses) and does not provide power for the reader to scan, this must be provided by an external power source.

When scanning, the reader draws 80mAh per hour and you can therefore calculate how much external battery power you need. For example, using 6x rechargeable NiMH AA batteries (2550mAh) will power the reader for 31 hours continuously (2550/80 = 310). The unit can last longer if it is programmed to scan during certain hours of the day only. Alternative battery sources, such as sealed lead acid (SLA) batteries can be used also.

The appropriate power source must be soldered to the PCB. Below is an example using a 6xAA battery pack (Fig. 2). The red wire is attached to the positive terminal, and the black wire is attached to the negative terminal (Fig. 2). In this example, thread the exposed wire through the PCB and bend the wire to hold them in place. On the back of the PCB, use the soldering iron to heat the metal coating of the hole that the wire has come through and add some solder. The solder should not form a large spherical blob (or it will easily disconnect later), it should ideally look like a cone from the base of the PCB, thinning up the wire. If it appears spherical, hold the

soldering iron to the PCB and the cable, heating both parts, until the solder bonds to them in a more conical shape. Any excess cable poking out can be clipped off with wire clippers.

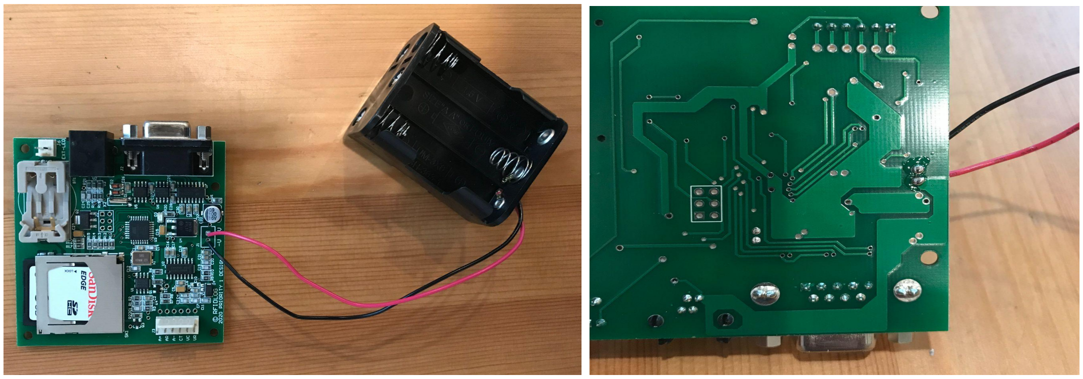

Figure 2. *External battery pack (left) soldered directly to the back of the PCB (right).*

2. Attaching the antenna

To attach the antenna to the PCB, Priority 1 Designs provides crimps header crimps so that the antenna can easily be attached/disconnected from the PCB. You can directly attach these to the copper wire of the antenna, or alternatively attach another cable in between (to the antenna to be further away from the PCB). Below is an example where a multicore figure-8 cable was used to connect the antenna to the PCB.

Peel apart the two strands from the end of the figure-8 cable. If you find it easier, you can first ‘tin’ the ends of the wire by melting some solder to the exposed strands (Fig.3a). Attach a crimp to each end of the wire where the first jaws of the crimp are around the entire cable, and the second jaws surround the exposed wire. Close the teeth shut using a crimping tool or a small pair of pliers. Using the soldering iron, heat the exposed wire in the crimp and add some solder. Ensure that the crimp is securely attached and soldered to the wire (which will later experience pulling pressure when inside the header) (Fig. 3b). The crimped ends can now be attached into the header.

(a)

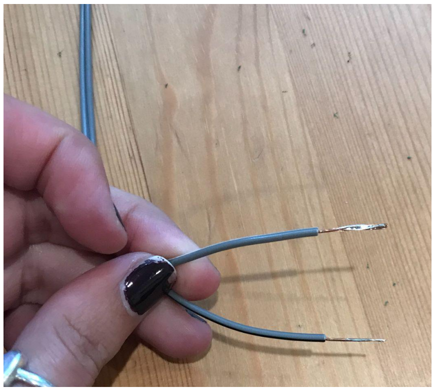

(b)

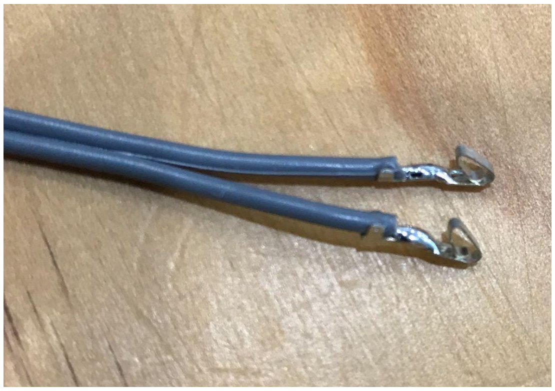

Figure 3. *Figure 8 cable tinned with solder (a) and then attached to crimp pins (b)*

The two copper wires coming from the end of the antenna will each be joined to a strand of the figure 8 wire. Be careful to note that the end of the copper wire appears more silver, as this part of the wire is not covered by insulation. You must connect the exposed ends to the figure-8 cable in order to make a strong enough circuit connection. If the exposed (silver) area is not big enough, or clipped off, then you can remove the insulation by sanding (using a fine sandpaper or nail file) or running the wire through hot solder, until the ends appear silver. If this is not done, the wire will not attach to the solder properly (e.g. Fig. 4) and will not provide a strong enough connection.

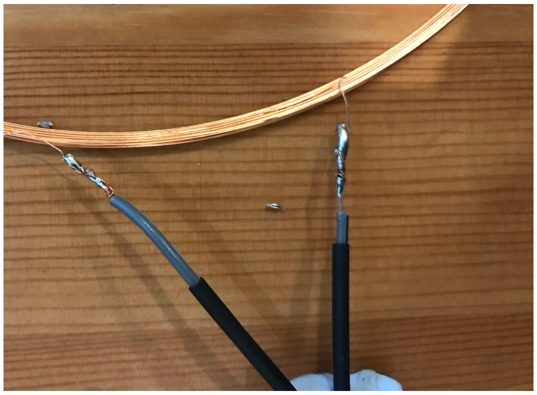

Figure 4. *Bad connection between ends of antenna wires (still covered in copper coloured insulation) and figure 8 cable.*

When the wires from the antenna are exposed, thread a small section of heatshrink onto the figure-8 cable, and then wrap each of the antenna ends around the end of the figure-8 cable ends. Twist the wires together ensuring the two cables make substantial contact. Solder the connection together (Fig. 5).

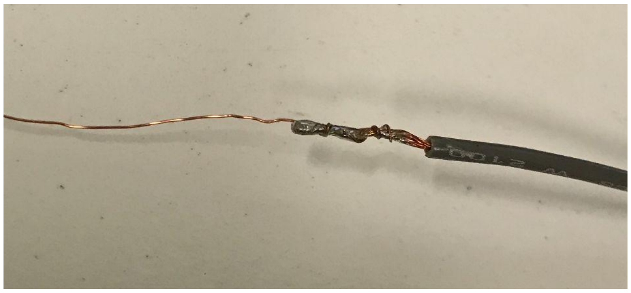

Figure 5. *Secure soldered connection between the figure 8 cable and RFID antenna.*

Then pull the heatshrink back over the connection, and apply heat from a flame gun to tighten and secure the connection. (Fig. 6)

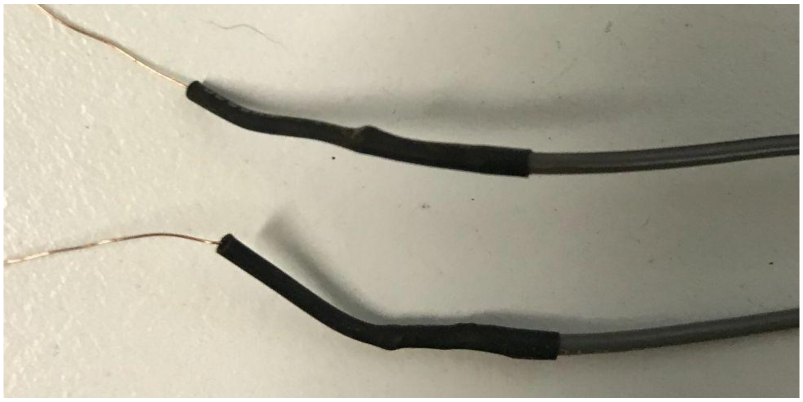

Figure 6. *Heat shrink enclosing the connection between the figure 8 cable and RFID antenna.*

To ensure the antenna is adequately connected, you can attach the header (with the crimps inside) to the PCB and plug the PCB into a computer using a D9 RS232 cable. If the cable does not connect to the app, ensure the correct port is selected (shows in Device manager) and ensure that the driver is installed for the specific cable. Open the Priority One app and click the "MOF" (measure unit operating frequency) button (Fig. 7). The frequency should return a number close to 124kHz. If the number returns more than ~5kHz below that, or 000, then the antenna does not have a good enough connection. Repeat the steps above (check the soldering connection and the crimp pins).

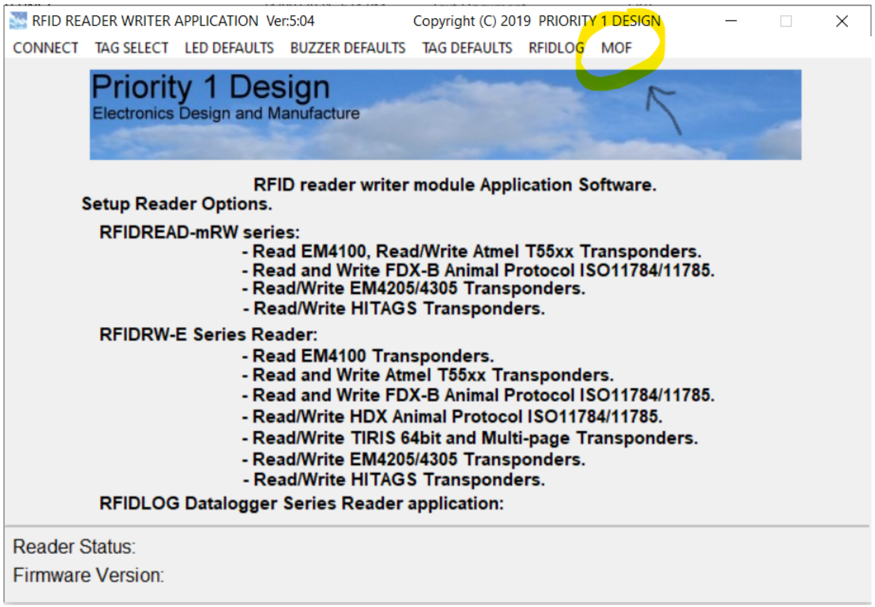

Figure 7. *Priority 1 Design RFID application interface.*

Once you are confident that the connection is strong, pull the heatshrink over then join and apply the heat gun to tighten it.

3. Waterproof housing

To protect the loggers from the elements when deployed in the field, we recommend enclosing the readers in water-proof housing. In the example below, we used plastic sandwich containers with small holes drilled in them (sealed with silicone).

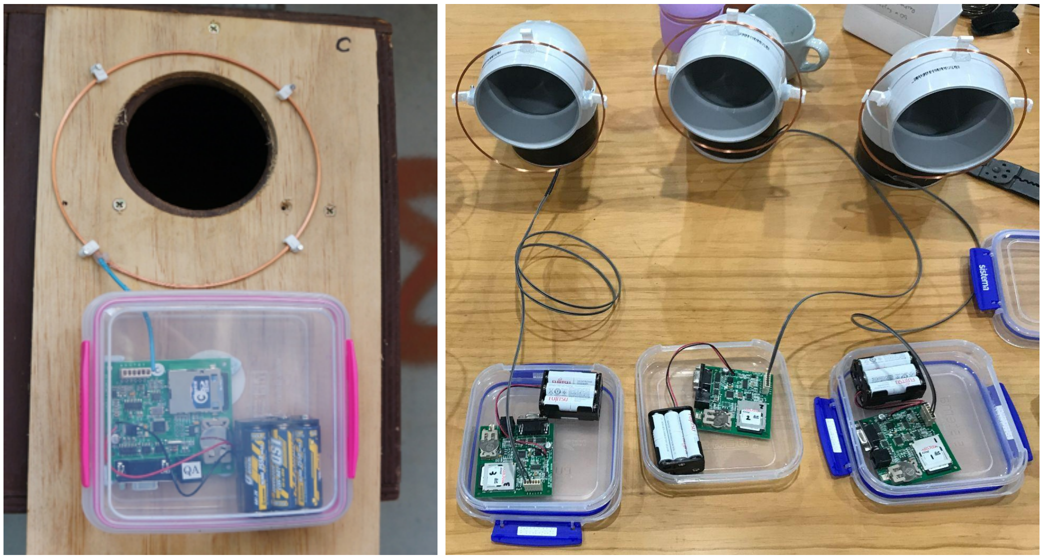

Figure 8. *Plastic sandwich containers as weather-proof housing for RFID loggers.*
