# Supplementary material for: Affordable RFID loggers for monitoring animal movement, activity, and behaviour
Source: PLoS One. 2022 Oct 27;17(10):e0276388. doi: 10.1371/journal.pone.0276388 (PMC9612574; doi:10.1371/journal.pone.0276388)
Supplement: S2 File — (PDF) [file pone.0276388.s003.pdf]

Power Analysis for RFID loggers

From Harrison & Kelly (2022)

Power calculations for balanced one-way analysis of variance tests where k is the number of groups, n is the number of samples in each, and f is the effect s i ze. H ere w e c hoose a c onservative e ffect si ze of 0. 3 (Cohen 1988, pg. 25).

With 2 commercial RFID loggers over 3 three nights, one could record 6 data points, 3 per group.

```
##commercial RFID loggers:
pwr.anova.test(k =2 , n =3 , f =0.30 , sig.level =0.05 , power = NULL)
```

```
##
##      Balanced one-way analysis of variance power calculation
##
##               k = 2
##               n = 3
##               f = 0.3
##       sig.level = 0.05
##           power = 0.08877466
##
## NOTE: n is number in each group
```

Using the same budget, one can purchase the components for 27 self-built RFID loggers, leading to 81 data points over three nights, 40 per group.

```
##self-built RFID loggers:
pwr.anova.test(k =2 , n =40 , f =0.30 , sig.level =0.05 , power = NULL)
```

```
##
##      Balanced one-way analysis of variance power calculation
##
##               k = 2
##               n = 40
##               f = 0.3
##       sig.level = 0.05
##           power = 0.7549516
##
## NOTE: n is number in each group
```

By using self-built RFID loggers, sample size increases from 6 to 81, and the power to detect a subtle difference in emergence time between two groups (e.g. sexes) increases from 0.09 to 0.75.

References

Champely (2020). pwr: Basic Functions for Power Analysis. R package version 1.3-0. https://CRAN.R-project.org/package=pwr

Cohen, J. (1988). Statistical Power Analysis for the Behavioral Sciences (2nd ed.). Lawrence Erlbaum Associates, New York.
