# Supplementary material for: Affordable RFID loggers for monitoring animal movement, activity, and behaviour
Source: PLoS One. 2022 Oct 27;17(10):e0276388. doi: 10.1371/journal.pone.0276388 (PMC9612574; doi:10.1371/journal.pone.0276388)
Supplement: S3 File — (PDF) [file pone.0276388.s004.pdf]

RFIDLOG

Dual animal tag data logger with external antenna and SD card storage.

The RFIDLOG is a RFID reader module capable of reading animal tags using the FDX-B and HDX protocols as described by ISO11784/11785. It features a time and date circuit and SD card interface to allow scanned codes to be recorded with a time stamp for research and general data logging applications. Additionally it may be operated in dual mode with an auxiliary reader connected for data logging of scanned codes at two connected locations.

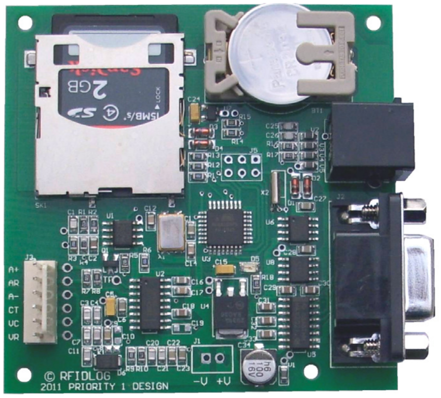

- **Suitable with the following transponders:**
  Read FDX-B and HDX Protocol as defined in ISO11784/11785 for animal identification.
- **Serial RS232 Output and Input on a DB9 connector.**
- **SD card storage interface, using Fat32 file system.**
- **Clock calendar circuit for time and date mark.**
- **Small unit size: 74.3mm x 74.3mm (RFIDLOG), and 46.1mm x 38.1mm (RFIDLOG-AUX)**
- **External RFID Coil Antenna for greater versatility.**
- **6.0V-13.2V input supply**

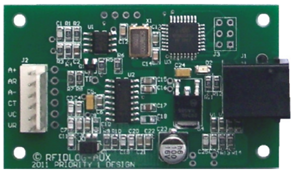

Description.

The RFIDLOG is designed to read FDX-B and HDX transponders as defined in ISO11784/11785. The main unit may be used on its own, or optionally connected to its auxiliary unit RFIDLOG-AUX to allow scanning to take place at two locations simultaneously. Codes scanned at either unit are stored along with reader designation, and time and date information into a SD memory card. The RFIDLOG employs standard Fat32 file storage which allow users to simply remove the SD card and insert into any standard SD card reader connected to a PC. The stored data file appears as an ordinary text file and can be manipulated as required.

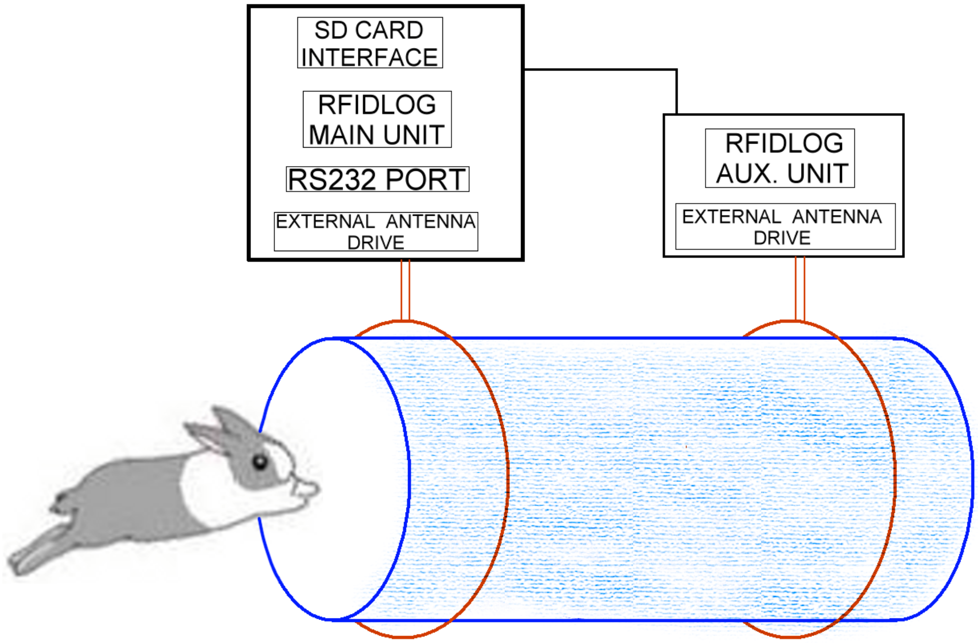

Fig 1. Dual reader configuration. Main unit and Auxiliary unit connected.

Priority 1 Design
Suite 6, 250 Gore Street
Fitzroy, 3065
Melbourne, Australia.

Document No: MAN_RFIDLOG
Revision: B
Modified: August 16, 2012
Reader firmware version: 404

Connector Pin Description:
The RFIDLOG main unit is powered by a DC supply at Lines –V, and V+ on the terminal pad area J1. Command and data information is available on a DB9 connector J2 as standard RS232 protocol. An external antenna is required to be connected to connector J3 for the rfid reader to be operational. A RJ11-4 connector is available at J4 for connecting the Auxiliary rfid reader if required.

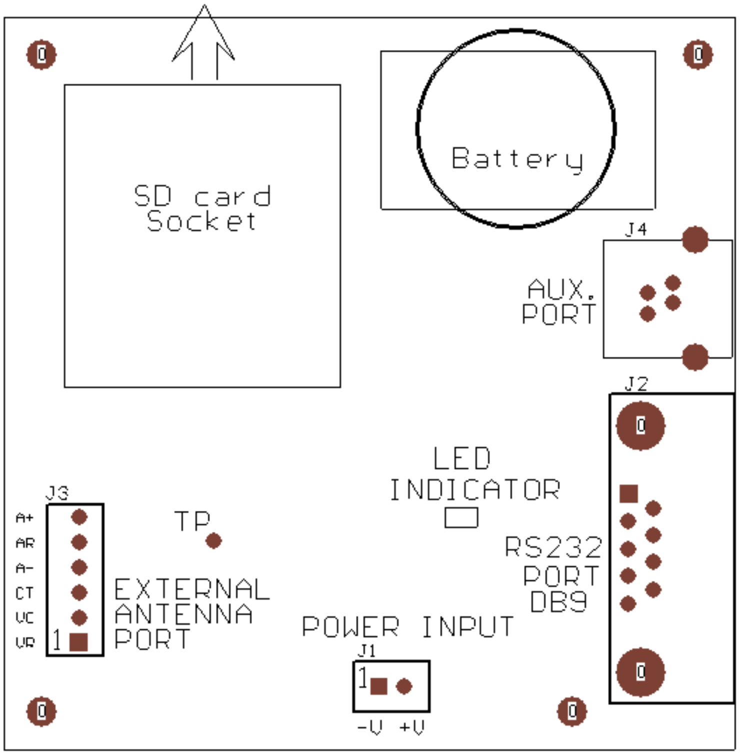

Fig 2. Main Unit connector diagram.

Connector J1 interface lines are described in Table 1.

Pin.	Label.	Description.
1	-V	System Ground line.
2	+V	System power input line. +6.0 to 13.2V DC

Table 1. J1 connector interface.

Connector J2, RS232 signal port lines are described in Table 2.

Pin.	Description.
2	Uart TX RS232 output. RFID data and command responses from the RFIDLOG are sent on this line
3	Uart RX RS232 input. Commands to the RFIDLOG unit are sent on this line.
5	Signal Ground line.

Table 2. J2 connector interface. RS232 signal port.

Connector J3, Main unit external antenna connections are described in Table 3.

Pin.	Label.	Description.
1	VR	Signal Sense point.
2	VC	An additional divisor capacitor may be added between VC and VR to maximize signal strength at VR.
3	CT	A tuning capacitor is attached between CT and VC (or A-) to tune the unit to the operating frequency.
4	A-	The return side of the antenna coil is connected here. Note that A- and VC are internally connected together.
5	AR	This line is the same as A+ but with the internal 22ohm series resistor bypassed.
6	A+	One side of the antenna coil is connected here, through a 22ohm on board series resistor.

Table 3. J3 External antenna port.

The RFIDLOG Auxiliary unit is an optional reader that can be connected to the main unit if dual scanning is required. Power and data communications is supplied via a RJ11-4 connector J1. An external antenna is required to be connected to connector J2 for the rfid reader to be operational. A standard 4 core RJ11 cable is used to connect the Auxiliary unit to the Main unit.

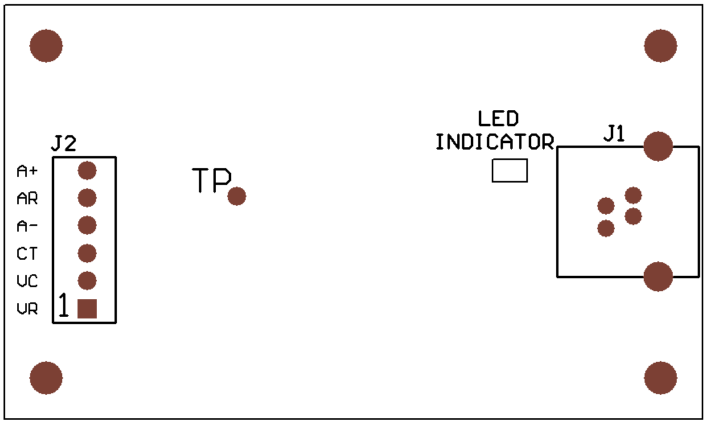

Fig 3. Auxiliary unit connector diagram.

Connector J2, Auxiliary unit external antenna connections are described in Table 4.

Pin.	Label.	Description.
1	VR	Signal Sense point.
2	VC	An additional divisor capacitor may be added between VC and VR to maximize signal strength at VR.
3	CT	A tuning capacitor is attached between CT and VC (or A-) to tune the unit to the operating frequency.
4	A-	The return side of the antenna coil is connected here. Note that A- and VC are internally connected together.
5	AR	This line is the same as A+ but with the internal 22ohm series resistor bypassed.
6	A+	One side of the antenna coil is connected here, through a 22ohm on board series resistor.

Table 4. J2 External antenna port.

Maximum ratings:

V+ to GND ....-0.3V to 13.2Vdc
RS232 signals ....……......... –25V to 25V
Operating Current Main unit ………….. 25mA (2)
Operating Current Auxiliary unit……….. 16mA (2)
Operating Current Dual + antennas.…….. 88mA
Operating Temperature Range ....0° C to 85°C
Storage Temperature Range ....0° C to 85°C
Maximum RFID antenna voltage ………….400Vpp

ABSOLUTE MAXIMUM RATINGS (1)

NOTE: (1) Stresses above those listed under “Absolute Maximum Ratings” may cause permanent damage to the device. Exposure to absolute maximum conditions for extended periods may affect unit reliability.
NOTE: (2) Excluding antenna current. May vary due to component variations

RFIDLOG operation.

The RFIDLOG unit can operate in stand alone fashion, or in tandem with the Auxiliary unit. In stand alone operation the main RFIDLOG unit continually scans for transponders. When one is detected a record entry is stored in the log file containing the time and date of detection, reader designation code, and identity code.

When the RFIDLOG auxiliary unit is attached both the main unit and auxiliary unit continually scan for transponders. When one is detected a record entry is stored in the log file on board the main unit. Record entries recorded by the Auxiliary unit carry the designation code "A", while records entered by the Main unit carry the designation code "M". Figure 4 contains an example of the RFIDLOG record file contents.

An on board LED on either unit will flash briefly to acknowledge that a transponder has been scanned.

```
110505174450 M  $3B500009D46A9
110505174501 A  $3B500009D46A9
110505182046 M  $07C000E3F09F8
110505182101 A  $07C000E3F09F8
110506120903 A  $3B500009D46A9
110506120906 M  $3B500009D46A9
```

Fig 4. Example record storage within the RFIDLOG.DAT file in SD memory card.

Records are stored inside a SD memory card which can be removed from the unit and replaced as needed. The RFIDLOG comes supplied with a 2 Gigabyte SD card as standard. Files are stored in Windows Fat32 file system format allowing the SD card to be read by any standard card reader.

A pre-formatted SD card must be used. When inserted the following files are automatically created if not already present.

RFIDLOG.CFG
RFIDLOG.DAT

RFIDLOG.CFG is a general configuration file used to preserve various program options. RFIDLOG.DAT is a text file which is used to store the accumulated records. New records are appended to older records if any are present.

The records are stored in the following format. 1 line per record, with each record being 31 characters long. The records contain a 12 digit time and date marker, a unit designator separated by spaces, the tag ID (15 characters decimal notation, or 13 character hexadecimal with $ sign indicator), and a carriage return as end of record marker.

eg.

101223152500 M 999000000001007<crn>   _M_ : Tag scanned in Main unit, ID in decimal notation
101223152600 A 999000000008888<crn>   _A_ : Tag scanned in Auxiliary unit
110506120906 M $3B500009D46A9<crn>   _M_ : Tag scanned in Main unit, ID in hexadecimal notation
a space and $ character precede a hexadecimal code.

The first 3 digits of a transponder code is always the Country code prefix, while the remaining characters are identity code values.

The log file may be downloaded via the serial port, or viewed directly from the SD card using a card reader. When the log file is viewed directly using a card reader the file format is as described above with the exception that a Line feed character is appended to the end of the line after the carriage return.

The time and date segments of the record are set up as Year, Month, Day, hours, minutes, seconds.
Eg. 101223152500   2010, December 23, 03:25 PM and 00 seconds

RFIDLOG command description.

Various commands and parameter data are sent to the RFIDLOG via the RS232 serial port. Commands sent to the reader consist of simple ASCII strings terminated with a carriage return. The reader will then process the command and respond by transmitting data or status information serially back through the serial port.

The RFIDLOG commands as summarized in Table 5. RFIDLOG Command summary.

Command Description	Serial Command Code.
TIME SET	TSxxxxxxxxxxxx<crn>
READ TIME	RTM<crn>
READ LOG FILE	RLF<crn>
CLEAR LOG FILE	CLF<crn>
UPDATE LOG FILE	ULF<crn>
READ RECORD SIZE	RRScrn>
SET READER DE-ACTIVE	SRD<crn>
SET READER ACTIVE	SRA<crn>
SET FORMAT HEXADECIMAL	SFH<crn>
SET FORMAT DECIMAL	SFD<crn>
SET OUTPUT MODE	SOx<crn>
SET INTERVAL MODE (timed mode command)	SIMxxxxyyyy<crn>
SET BEGIN TIME (timed mode command)	SBx<crn>
SET END TIME (timed mode command)	SEx<crn>
SET CURRENT DAY (timed mode command)	SDx<crn>
READ TIMED PARAMETERS	RTP<crn>
MEASURE OPERATING FREQUENCY (main unit only)	MOF<CRN>
READ FIRMWARE VERSION CODE	VER<crn>

Table 5. RFIDLOG Command Summary..

Time Set Command.

The RFIDLOG contains a clock calendar circuit and battery backup for retaining time and date information during power down cycles. The correct time and date needs to be initialized so that the time and date marker added to the record is correct. It is also recommended that regular time initialization be performed as any minor clock timing inaccuracies that may exist will accumulate over time.

The command protocol for which is shown here, along with the available responses

Protocol: **TSyymmddhhmnss<crn>** ,where <crn> is $0D carriage return, and yymmddhhmnss denotes Year, Month, Day, hours, minutes, seconds

Command Protocol Example	Response.	Description.
**TS101223152500<crn>**	**OK<crn>**	**Date: 2010 Dec 23**<br>**Time 3:25:00PM is set**

Read Time Command.

The time and date set within the RFIDLOG can be read with this command.

The command protocol for which is shown here, along with the available responses

Protocol: **RTM<crn>** ,where <crn> is $0D carriage return.

Command Protocol Example	Response.	Description.
**RTM<crn>**	**yymmddhhmnss cd<crn>**	yymmddhhmnss denotes Year, Month, Day, ,hours, minutes, seconds. cd denotes the current day as used in interval mode (01-07. 1= Monday).

Read Log File Command.
The log file records can be read via the RS232 port using this command. Once this command is issued records will be sent continuously until all records are sent or an “ESC” character is sent by the application via the RS232 port.

Protocol: **RLF<crn>** ,where <crn> is $0D carriage return.

Command Protocol Example	Response.	Description.
**RLF<crn>**	101223152500 M 999000000001007<crn><br>101223152600 A 999000000008888<crn><br>.<br>.<br>**OK<crn>**	**Records download**

Clear Log File Command.
This command is used to clear the log file of records.
The command protocol for which is shown here, along with the available responses.

Protocol: **CLF<crn>** ,where <crn> is $0D carriage return.

Command Protocol Example	Response.	Description.
**CLF<crn>**	**OK<crn>**	**Long file is cleared of records.**

Update Log File Command.
The record length that is required to be stored when a transponder is scanned is 32 characters long. However a characteristic of SD memory cards is that data is written in blocks of 512 characters at a time. They are also subject to a limited number of Write cycles. In order to extend the life span of the SD card the RFIDLOG unit stores records in a FRAM memory device and will only update the SD card when 16 records have been created. The FRAM memory device is capable of preserving its memory without battery backup even through a power loss.
As a result of this mechanism being employed the records stored in SD card may not always be up to date as some may exist in FRAM memory. The SD memory card can be updated with the latest records under the following conditions:

1. When the RFIDLOG is turned on a SD card update will occur.
2. If the SD card is removed and replaced while power is applied a SD card update will occur.
3. If the Update Log File command is issued via the RS232 interface a SD card update will occur.

Protocol: **ULF<crn>** ,where <crn> is $0D carriage return.

Command Protocol Example	Response.	Description.
**ULF<crn>**	**OK<crn>**	**SD memory card is updated with latest records stored in FRAM memory.**

Read Record Size Command.
This command is used to learn the number of records currently stored in the log file including those stored in FRAM memory

Protocol: **RRS<crn>** ,where <crn> is $0D carriage return.

Command Protocol Example	Response.	Description.
**RRS<crn>**	**xxxxxxxx(y)**	**Where xxxxxxxx is the total number of records available, and y is the number of records still in FRAM memory.**

Note: Response is in Hexadecimal notation.
Eg.

**RRS<crn>**	**000004D3(3)**<br>**.**<br>**.**<br>**OK<crn>**	**there are 1235 records available, 3 of which are in FRAM storage.**

Set Reader De-active Command.
This command is used to turn off the RF field emitted by the RFIDLOG. Tags can not be scanned while in this mode. This mode is used to prevent the RF field of the reader from interfering with other readers in the vicinity.
The command protocol for which is shown here, along with the available responses.

Protocol: **SRD<crn>** ,where <crn> is $0D carriage return.

Command Protocol Example	Response.	Description.
**SRD<crn>**	**OK<crn>**	**RF field is turned off**

On power up the RFIDLOG will default to Reader Active mode.

Set Reader Active Command.
This command is used to turn on the RF field emitted by the RFIDLOG after it has been turned off using the Set Reader De-active Command. Tag scanning will resume in this mode.
The command protocol for which is shown here, along with the available responses.

Protocol: **SRA<crn>** ,where <crn> is $0D carriage return.

Command Protocol Example	Response.	Description.
**SRA<crn>**	**OK<crn>**	**RF field is turned on.**

Set Format Hexadecimal command.
The identity code of the transponder can be stored in either decimal, or hexadecimal notation. To store records in Hexadecimal notion the Set Format Hexadecimal command is used. The command only needs to be issued once as the RFIDLOG will retain this parameter once set. When storing ID codes in Hexadecimal format a space and $ character are used as a prefix to the ID code stored in the record. This is to both preserve the 15 character length in the record string allocated to the ID code, as well as to allow the user to distinguish between records stored in Decimal and Hexadecimal format.
The command protocol for which is shown here, along with the available responses.

Protocol: **SFH<crn>** ,where <crn> is $0D carriage return.

Command Protocol Example	Response.	Description.
**SFH<crn>**	**OK<crn>**	**Store ID in Hexadecimal format.**

Set Format Decimal command.
The identity code of the transponder can be stored in either decimal, or hexadecimal notation. To store records on Decimal notion the Set Format Decimal command is used. The command only needs to be issued once as the RFIDLOG will retain this parameter once set.
The command protocol for which is shown here, along with the available responses.

Protocol: **SFD<crn>** ,where <crn> is $0D carriage return.

Command Protocol Example	Response.	Description.
**SFD<crn>**	**OK<crn>**	**Store ID in Decimal format.**

Set Output Mode.
This command is used to control whether the scanned code is transmitted via the serial port before being stored in SD card memory. The format of the output will depend on whether hexadecimal or decimal format has been set. This command only needs to be issued once as the mode of operation is retained even when the unit is turned off.

The command protocol for which is shown here, along with the available responses.

Protocol: **SOx<crn>** ,where x = 0 for disable output to serial port,
x = 1 for enable output to serial port,
<crn> is $0D carriage return.

Command Protocol Example	Response.	Description.
**SO1<crn>**	**OK<crn>**	**Unit will now output the scanned code via the serial port before storing to SD card.**
**SO0<crn>**	**OK<crn>**	**Unit will not output the scanned code via the serial port before storing to SD card.**

Set Interval Mode.
In some situations it is preferred that the RFIDLOG scans for a period of time and then stops scanning for another period of time, before repeating the scan cycle. This mode helps reduce power consumption during periods where scanning is not required.

The command protocol for which is shown here, along with the available responses.

Protocol: **SIMxxxxyyyy<crn**> ,where xxxx = Field on scanning duration in seconds, yyyy = Field Off Not scanning duration in seconds. xxxx, and yyyy are 4 hexadecimal characters each. When either xxxx, or yyyy = 0 Interval mode is disabled
<crn> is $0D carriage return.

**Command Protocol Example**	**Response.**	**Description.**
**SIM0005001B<crn>**	**OK<crn>**	**Unit scans 5 seconds on, 27 seconds off.**

Timed Mode Commands.
In addition to scanning for a preset time interval (see SIM command above) the RFIDLOG can also be set to turn on and off at a specific time in each of the 7 days of the week. There are 3 commands that allow the setting of these parameters, shown below.

Set Begin Time.
The Set Begin time sets the time in the specified day of the week when scanning will begin.

The command protocol for which is shown here, along with the available responses.

Protocol: **SBxhrmn<crn**> ,where x = 1-7 denoting the day of the week. 1 = Monday, , hrmn = 4 ascii characters representing hours and minutes in 24hr mode. Eg 1305 = 1:05PM.
<crn> is $0D carriage return.

**Command Protocol Example**	**Response.**	**Description.**
**SB31305<crn>**	**OK<crn>**	**Unit activates on Wednesday 1:05PM**

Set End Time.
The Set End time sets the time in the specified day of the week when scanning will end.
The command protocol for which is shown here, along with the available responses.

Protocol: **SExhrmn<crn**> ,where x = 1-7 denoting the day of the week. 1 = Monday, , hrmn = 4 ascii characters representing hours and minutes in 24hr mode. Eg 1305 = 1:05PM.
<crn> is $0D carriage return.

**Command Protocol Example**	**Response.**	**Description.**
**SE31625<crn>**	**OK<crn>**	**Unit deactivates on Wednesday 4:25PM**

The start and end times can be individually set for each of the 7 days of the week. When ever the Start time equals the End time the unit is assumed to be active the entire day. Whenever the Start time is greater than the End time the unit is assumed to be de-active the entire day. Start and end times must be within the same day.

Set Day command.
The RFIDLOG has no means to determine what the current day of the week is. For this reason Monday is arbitrarily defined as day 1, with the days coming in sequence ending with day 7 being Sunday.
The current day needs to be defined when setting up the Timed mode parameters which means that this command should be sent in conjunction with the Set start time, and Set end time commands.

The command protocol for which is shown here, along with the available responses.

Protocol: **SDx<crn**> ,where x = 1-7 denoting the current day of the week. 1 = Monday
<crn> is $0D carriage return.

Command Protocol Example	Response.	Description.
**SB31305<crn>**	**OK<crn>**	**Unit activates on Wednesday 1:05PM**
**SE31625<crn>**	**OK<crn>**	**Unit deactivates on Wednesday 4:25PM**
**SD2<crn>**	**OK<crn>**	**Today is Tuesday.**

Read Timed Parameters command.
The current timed mode parameters set using the above commands can be read from the RFIDLOG using this command.

The command protocol for which is shown here, along with the available responses.

Protocol: **RTP<crn**> , <crn> is $0D carriage return.

Response protocol:
aaaa AAAA bbbb BBBB cccc CCCC dddd DDDD eeee EEEE ffff FFFF gggg GGGG hh iiii jjjj<crn>
OK<crn>

Where

aaaa = start scan time Monday, AAAA = stop scan time Monday
bbbb = start scan time Tuesday, BBBB = stop scan time Tuesday,
……
gggg = start scan time Sunday, GGGG = stop scan time Sunday,
hh = current day.
iiii = Scanning Interval on period in seconds (hexadecimal value)
jjjj = Scanning Interval off period in seconds (hexadecimal value)

Measure Operating frequency command.
The RFIDLOG allows for an external antenna to be connected. For proper operation the specification of this antenna should be chosen in conjunction with the tuning capacitors so that the circuit operates at 134.4khz for optimal read range. To assist in tuning your antenna the Measure Operating frequency command may be used. This will cause the RFIDLOG to take a measurement and report the operating frequency of the connected antenna.

Protocol: **MOF<crn**> ,<crn> is $0D carriage return.

Command Protocol Example	Response.	Description.
**MOF<crn>**	**134.2<crn>**	**Measured frequency = 134.2 kHz**

This operation will require 1 second for the measurement to be taken and a response issued.

READ FIRMWARE VERSION CODE.
This command is used to read the firmware version number of the reader.

Protocol: **VER<crn>** , crn> is $0D carriage return.

Eg.

**Command Protocol**	**Response.**	**Description.**
**VER<crn>**	**404<crn>**	**Current Version number.**

Error Codes and Status Description.

The RFIDLOG unit will respond to every command with either the requested data, or one of these status strings summarized here.

ERROR AND STATUS CODES.	DESCRIPTION.
?0<crn>	Command not understood.
?1<crn>	Reserved
?2<crn>	SD memory card file error.
OK<crn>	Function Performed Successfully.

Table 6. Error Codes and Status Description summary.

Serial protocol description:
The protocol for the Serial Input and Output lines is 9600 Baud, 8 data bits, 1 stop bit, no parity.

Operating frequency:

In a RFID reader the antenna is a simple inductor, typically in the form of a coil. When an inductor is coupled to a capacitor and driven by an alternating signal the amplitude of the voltage across the inductor varies depending on the values of the drive frequency, the inductor, and the capacitor. This tuned circuit is illustrated in Figure 5. When the frequency reaches a critical value the voltage across the inductor reaches a maximum value which can be many times the amplitude of the driving voltage. This is known as the resonant frequency. RFID readers typically operate at this resonant frequency to maximize the power transferred to the antenna.

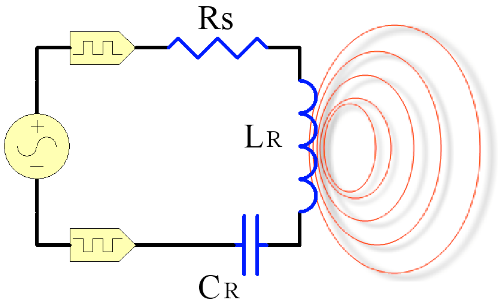

Where L_R = Inductance of the antenna.
C_R = Value of Tuning capacitor.
Rs = Series resistance of the antenna.

Figure 5. Basic tuned circuit

The resonant frequency of a tuned circuit can be found using the following formula.

Equation 1.

$$f_0 = \frac{1}{2 \times \pi \times \sqrt{L_R \times C_R}}$$

Where fo = the operating frequency
L_R = is the antenna inductance.
C_R = value of the tuning capacitor

The antenna drive on the RFIDLOG uses a self adaptive carrier frequency circuit to locate the antenna resonant frequency. This means that the antenna drive circuit will automatically tune the operating frequency to the antenna and tuning capacitor being used. This maximizes the power transferred to the antenna and increases read range. The drive circuit can operate anywhere in the range from 100 to 150 kHz depending on the values of L_R and C_R.

As the RFIDLOG unit is typically used to scan for animal transponders the frequency of operation should be tuned to 134.4khz.

Connecting the antenna:

The RFIDLOG has a series of terminals for antenna connection and configuration. These connection points are designated A+, AR, A-, CT, VC, and VR. The current operating frequency can be measured by using the MOF command described earlier in this document. Figure 6 illustrates the antenna drive circuit with a typical antenna connection.

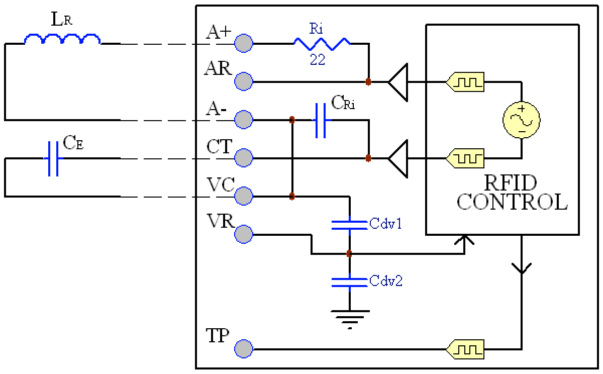

Figure 6. Antenna Drive Interface

This unit is designed to be connected to our standard RFIDCOIL-160A antenna but can easily be used with other coil antennas. Internal tuning capacitors Cri and Series Resistor Ri preset the unit to operate at 134Khz when used in conjunction with the RFIDCOIL-160A to minimize external components. When used with any other antenna coil a tuning capacitor is required to set the units operating frequency which is typically 134Khz.

As the voltage on a RFID antenna can be much larger than the input circuitry can accept, the voltage being sensed at the antenna is reduced by means of a simple capacitor divider circuit, shown in Figure 6 as Cdv1, and Cdv2. These values are preset to accept a maximum antenna voltage of 350V peak to peak..
The peak to peak voltage that will develop across the antenna coil is determined by the formula:

Equation 2. $$Va(p.p) = \frac{2}{fo \times C_R \times (Rt + 6)}$$

Where fo = the operating frequency.
C_R = is total value of tuning capacitor.
Rt = sum of any external series resistance and internal antenna resistance.

Also the peak current through the antenna can be determined by:

Equation 3. $$Ia(peak) = \frac{6.37}{(Rt + 6)}$$

Where Rt = sum of any external series resistance and internal antenna resistance.

The maximum voltage allowed to exist at the sensing point VR is 4.0V so the voltage divider circuit consisting of Cdv1 and Cdv2 reduces the large antenna voltage to a suitable level.
The maximum current (Ia) allowed through the antenna coil must not exceed 200mA.

Antenna connection pads are described in Table 7

Label.	Description.
A+	One side of the antenna coil is connected here, through a 22ohm on board series resistor.
AR	This line is the same as A+ but with the internal 22ohm series resistor bypassed. **(1)**
A-	The return side of the antenna coil is connected here. Note that A- and VC are internally connected together.
CT	A tuning capacitor is attached between CT and VC (or A-) to tune the unit to the operating frequency. **(2)**
VC	An additional divisor capacitor may be added between VC and VR to adjust signal strength at VR. **(3)**
VR	Signal Sense point.

Table 7. Antenna connection lines

Note: (1) This antenna connection is used instead of A+ whenever a series resistor other than the 22ohm internal resistance is required. A series resistor is used to limit the voltage developed across the antenna coil according to equation 2.

Note: (2) An internal tuning capacitor C_{Ri} of 532pF exists between A- and CT. Any capacitance added between CT and VC will add to this value. The tuning capacitor then become the sum of C_{Ri} and any externally applied capacitor. This value is then used in equation 1 with the antenna inductance to set the operating frequency of the unit.

Note: (3) The capacitor values of Cdv1 and Cdv2 are internally set at 11pF and 1nF respectively. Giving the unit a voltage reduction factor of 92. Any capacitor added across Cdv1 will alter this reduction value according to equation 3.

Equation 4.

$$\text{Voltage reduction factor} = \frac{\text{Cdv2} + \text{Cdv1} + \text{External capacitor (across VC andVR)}}{\text{Cdv1} + \text{External capacitor (across VC andVR)}}$$

$$= \frac{\text{1011pf} + \text{External capacitor (across VC andVR)}}{\text{11pf} + \text{External capacitor (across VC andVR)}}$$

Note: It is often required to fine tune a RFID antenna and tuning capacitor circuit to cater for any stray capacitance that occur in a circuit. To obtain a reading of the operating frequency the Measure Operating Frequency command (MOF) may be used.

Example circuit connections:

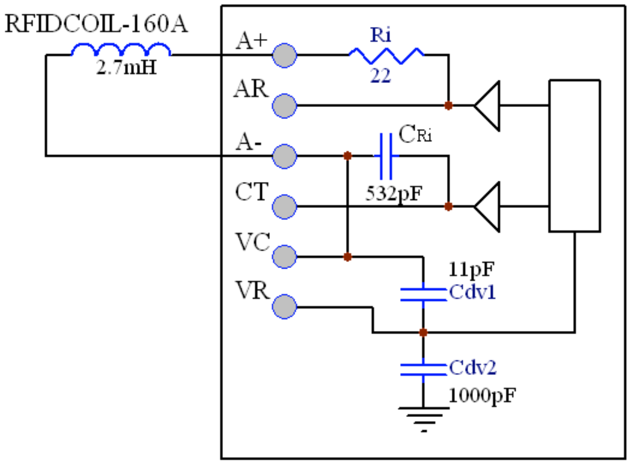

Antenna drive with RFIDCOIL-160A @ 134.4khz

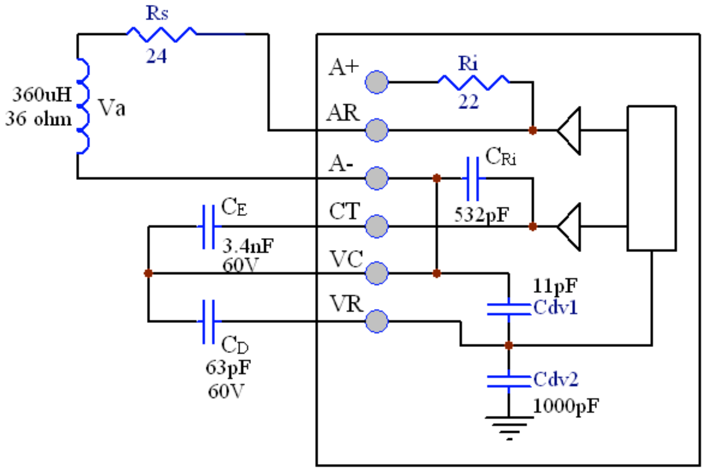

134khz generic coil design.

$$C_R = \frac{1}{L_R \times (2 \times \pi \times f_o)^2}$$ **(from Equation 1)**

$C_R = 3.9nF = C_E + C_{ri}$ Where $C_{ri} = 532pF$

Therefore $C_E \sim 3.4nF$

$$V_a = \frac{2}{f_o \times C_R \times (R_t + 6)} = \frac{2}{134000 \times 3.9nF \times 66} = 58V\ p.p$$

$$\text{Voltage at VR} = V_a \times \frac{(C_{dv1} + C_D)}{(C_{dv1} + C_D) + C_{dv2}}$$

$$(C_{dv1} + C_D) = \frac{C_{dv2} \times VR}{V_a - VR} = \frac{1000pF(4V)}{58V - 4V}$$

$(C_{dv1} + C_D) = 74pF$, $C_D \sim 63pF$

$$I_a = \frac{6.37}{(R_t + 6)} = 96.5mA \quad (<200\ \text{max})$$

RFID application software:
Our free RFID reader application program is designed to operate across the entire range of our RFID readers and can be downloaded from http://www.priority1design.com.au/download.html

An example screen shot of the RFIDLOG access screen is shown here:

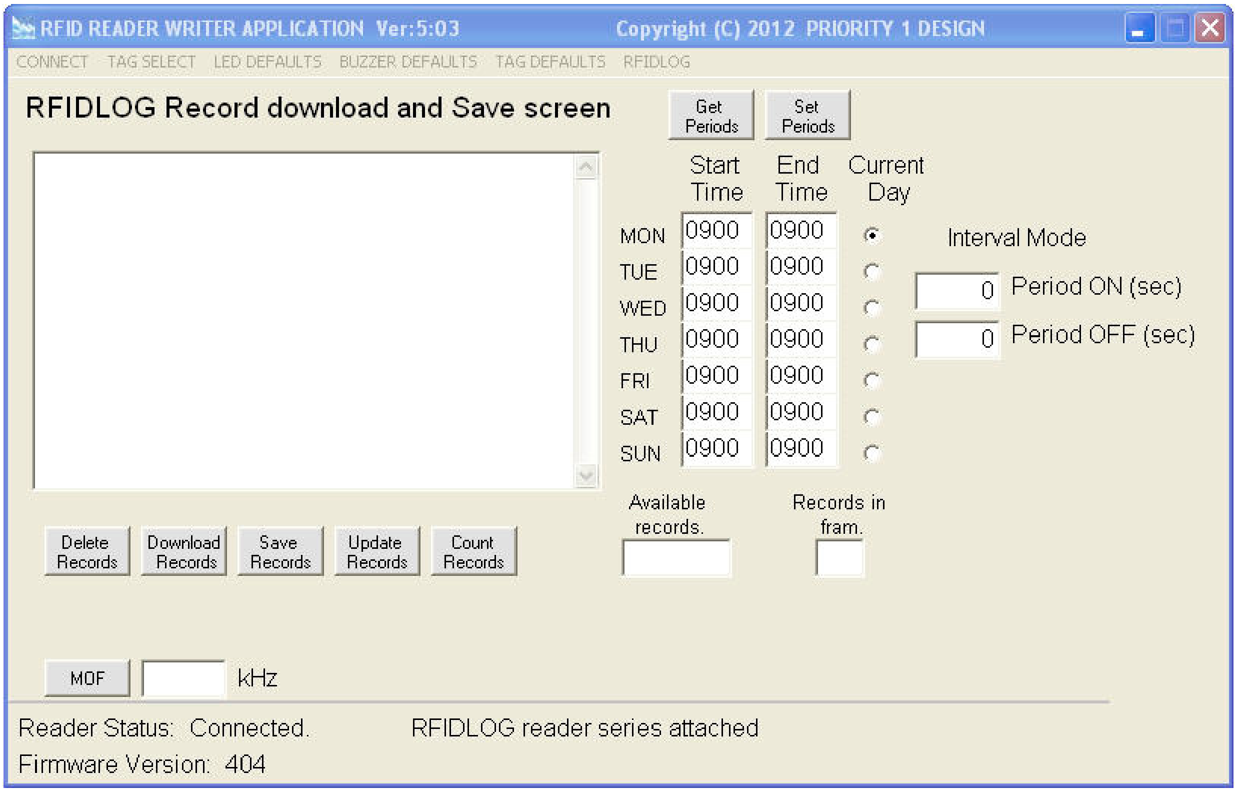

Dimensions:

RFIDLOG MAIN UNIT

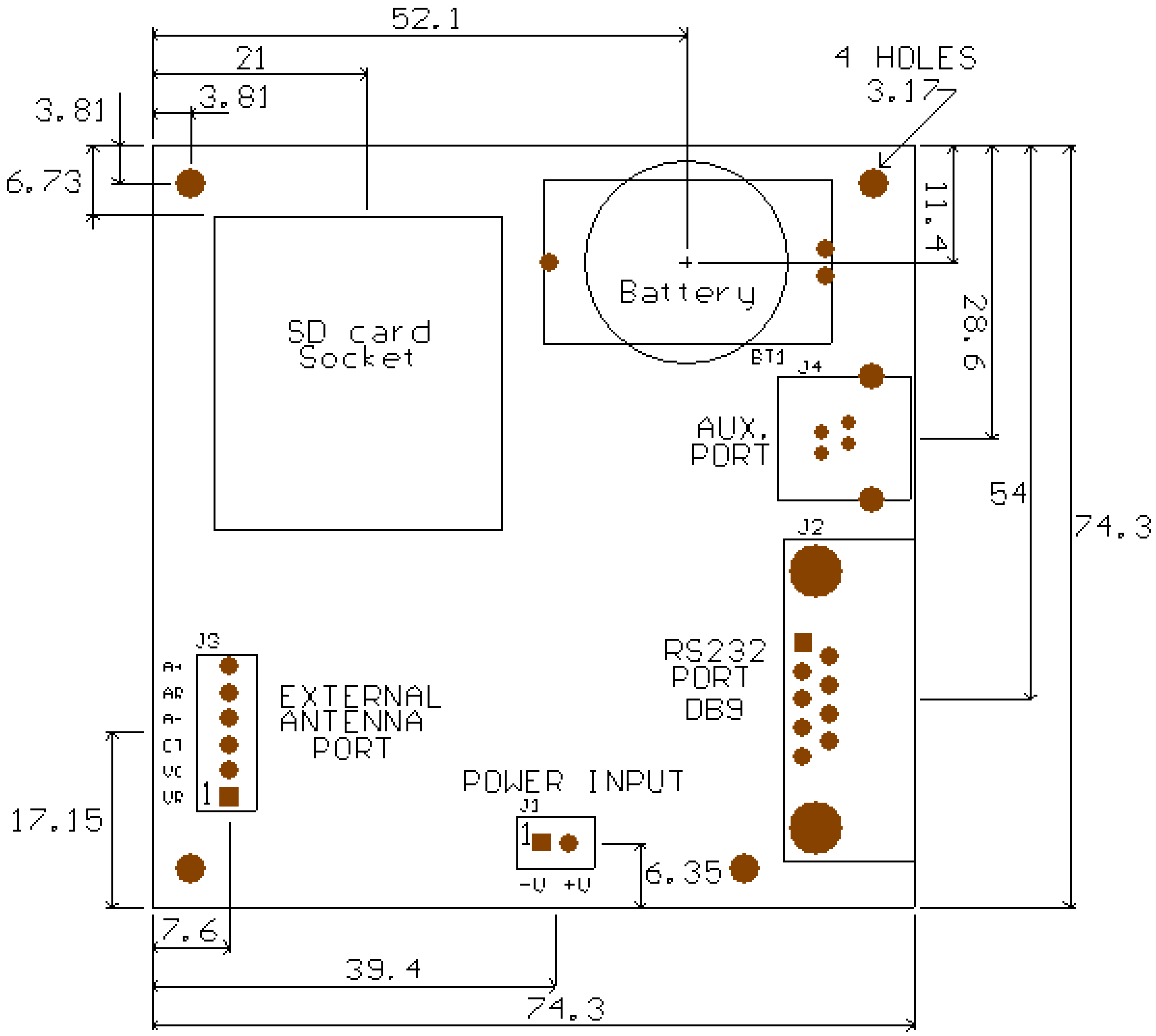

RFIDLOG AUXILIARY UNIT

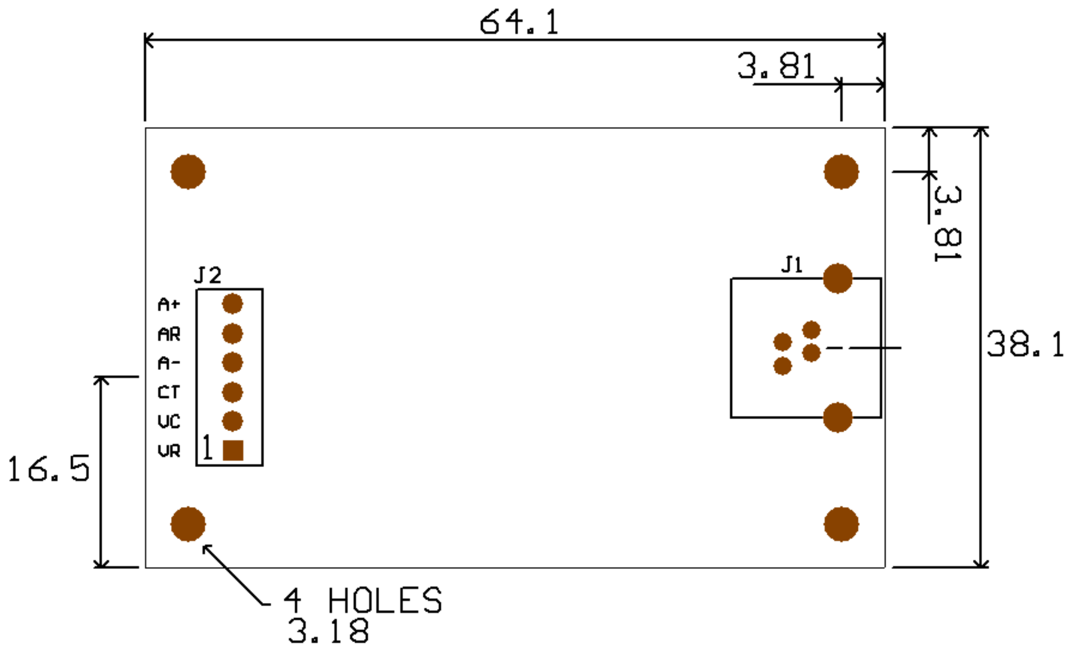

All dimensions in Millimeters.

The effects of noise on the RFIDLOG.
Reading a passive RFID transponder requires a sensitive receiver to read the data over the reading distance of the tag. As this requires detecting minute signals any electrical noise in the environment will affect read range. It is advisable to use a well regulated voltage supply free of additional noise otherwise read and write range can be drastically reduced. In addition the presence of noisy electronics such as high speed microprocessors, switch mode power supplies, or signals from other noisy sources such as USB interfaces will also impact the readers performance.
Additionally, if running the unit without the DB9 communication port connected to a PC the read range may be affected unless your power supply is either connected to earth, or is battery powered.

Disclaimers.

Priority 1 Design reserves the right to change specifications and prices at any time and without notice.
Priority 1 Design shall not be liable to recipients or any third party for any damages, including but not limited to personal injury, property damage, loss of profits, loss of use, interrupt of business or indirect, special incidental or consequential damages, of any kind, in connection with or arising out of the furnishing, performance or use of products supplied, or the technical data herein. No obligation or liability to recipient or any third party shall arise or flow out of Priority 1 Design rendering of technical or other services.

Life support — This product is not designed for use in life support appliances, devices, or systems where malfunction of these products can reasonably be expected to result in personal injury.
Priority 1 Design customers using or selling this product for use in such applications do so at their own risk and agree to fully indemnify Priority 1 Design for any damages resulting from such application.
