# Supplementary material for: Affordable RFID loggers for monitoring animal movement, activity, and behaviour
Source: PLoS One. 2022 Oct 27;17(10):e0276388. doi: 10.1371/journal.pone.0276388 (PMC9612574; doi:10.1371/journal.pone.0276388)
Supplement: S4 File — (DOCX) [file pone.0276388.s005.docx]

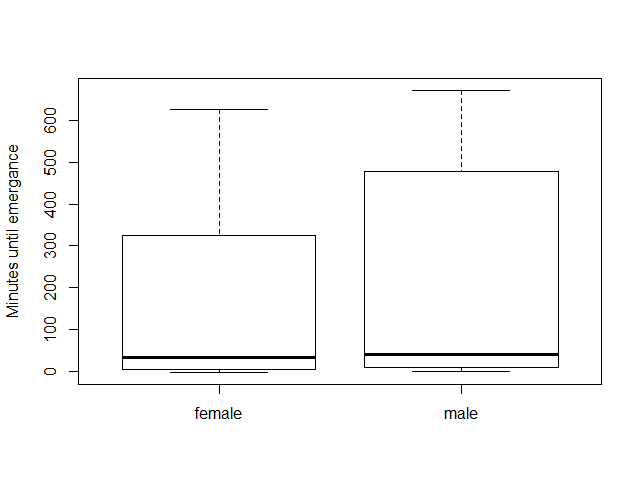
Figure S1. Boxplot of minutes until first emergence for male and female northern quolls during their first night in captivity.


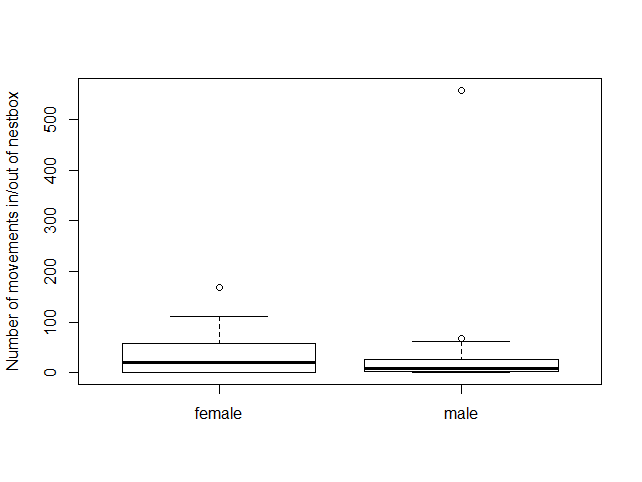
Figure S2. Boxplot showing number of records of northern quolls moving in or out of their nestboxes on the first night of captivity.
