# Supplementary material for: Affordable RFID loggers for monitoring animal movement, activity, and behaviour
Source: PLoS One. 2022 Oct 27;17(10):e0276388. doi: 10.1371/journal.pone.0276388 (PMC9612574; doi:10.1371/journal.pone.0276388)
Supplement: Attachment — Submitted filename: comments_to_author.docx [file pone.0276388.s006.docx]

Though the manuscript is interesting and the work reported is useful for researchers working in the area related to study of animal behaviour/habits. The manuscript needs improvisation on many points as explained under:

- The manuscript needs professional editing.
- Discussion part is too short, it needs to be elaborated using statistical techniques to draw detailed inferences about the species studied and performance of RFID loggers developed.
- It is not mentioned by the author that how many RFID loggers were used for Quenda study.
- Further, author should explain how and at which body part the RFID tags (PIT ) were implanted under the materials and method section.
- It is not explained, if there were any tags that didn’t work after deployment or there could be some Quenda and Quoll, which never entered the nest or antenna positioned.
- Only the data of Quenda study is correlated using the data of camera traps, the data of quoll study also needs to be explained in correlation with camera trap/other suitable techniques.
- References in the text needs to be arranged as per the format of the journal.
