# Supplementary material for: Affordable RFID loggers for monitoring animal movement, activity, and behaviour
Source: PLoS One. 2022 Oct 27;17(10):e0276388. doi: 10.1371/journal.pone.0276388 (PMC9612574; doi:10.1371/journal.pone.0276388)
Supplement: Attachment — Submitted filename: Response_to_reviewers.docx [file pone.0276388.s007.docx]

We thank our two anonymous reviewers for their comments. Please see responses to each comment below.

Reviewer #1.

Reviewer comment: Though the manuscript is interesting and the work reported is useful for researchers working in the area related to study of animal behaviour/habits. The manuscript needs improvisation on many points as explained under:
*Our response: Thank you for your suggestions. We have addressed them below.*

Reviewer comment: The manuscript needs professional editing.
*Our response: We have not sought professional editing, instead we have had a colleague proof-read the manuscript.*

Reviewer comment: Discussion part is too short, it needs to be elaborated using statistical techniques to draw detailed inferences about the species studied and performance of RFID loggers developed.
*Our response: We have now included an ANOVA in emergence time between male and female quolls (lines 162-163), an ANOVA in amount of activity between male and females quolls (lines 164-165), and estimates of within individual repeatability of both latency to emerge and foraging effort in quenda (142-144; 173-176). These results have been incorporated into the discussion (lines 197-200 and lines 223-226).*

Reviewer comment: It is not mentioned by the author that how many RFID loggers were used for Quenda study.
*Our response: At line 139 it reads “*We deployed 5 RFID loggers over 4 nights”

Reviewer comment: Further, author should explain how and at which body part the RFID tags (PIT) were implanted under the materials and method section.
*Our response: Thanks for this suggestion. At lines 94-95 we have added “*For use in small mammals, these PIT tags are inserted between the shoulder blades, on the back of the animal”

Reviewer comment: It is not explained, if there were any tags that didn’t work after deployment or there could be some Quenda and Quoll, which never entered the nest or antenna positioned.
*Our response: For the quoll study, we did not have camera traps paired with each RFID reader, so we do not have this information. For the quenda study, we have clarified that “individuals were correctly identified during 25 of these (80% of visits to feeding stations; not all visits resulted in an animal entering the station)” at lines 169-170.*

Reviewer comment: Only the data of Quenda study is correlated using the data of camera traps, the data of quoll study also needs to be explained in correlation with camera trap/other suitable techniques.
*Our response: Unfortunately, we did not have camera trap data for the quoll study so we are unable to correlate this information.*

Reviewer comment: References in the text needs to be arranged as per the format of the journal.
*Our response: We have reformatted the references to the style of the journal.*

Reviewer #2:

Reviewer comment: The work could be interesting. More technical discussion could be added as listed below.
*Our response: Thank you for your suggestions. We have addressed them below.*

Reviewer comment: 1. More RFIDs and RFID sensors should be reviewed and discussed e.g. J Zhang, etc., A review of passive RFID tag antenna-based sensors and systems for structural health monitoring applications, Sensors, 2017.
*Our response: Reviewing multiple RFID sensors as Zhang et al. have done is outside the scope of our study – we are intending to evaluate a cheaper RFID option, draw comparisons to commercial technology and describe potential applications. Zhang et al review a wide range of technologies, and in this study, we are mainly focussed on short-range RFID. We have specified this in the introduction at line 66 and have made reference to the Zhang et al. paper in a broader context at line 53.*

Reviewer comment: 2. The advantages of RFID monitoring systems and IOTs should be discussed.
*Our response: In the discussion, we describe some advantages of using RFID technology, specifically in regard to experimental design and sample size (lines 226-237). We have also now added lines discussing IOT and the potential application at lines 238-246 “Our RFID loggers remove the need for a human observer in the field, however, they still require some human effort to collect data (downloaded manually using the Priority 1 Software). Given the rapid development of sensor technology and the modular nature of these RFID loggers, it may be possible to connect these sensors to a mobile network, where data can be received straight into the cloud (the concept of Internet Of Things; IOT [9]. Though this was outside the scope of this particular study, we recommend futures studies considering our RFID loggers take the time to investigate the potential of IOT to increase efficiency in data collection through automation. Such an advancement also opens up many avenues for further application, such as remote trapping of targeted individuals, or access-limited nest boxes based on individual identity.”.*

Reviewer comment: 3. More quantitative data in section 3 are expected.
*Our response: We have now included an ANOVA in emergence time between male and female quolls (lines 162-163), an ANOVA in amount of activity between male and females quolls (lines 164-165), and estimates of within individual repeatability of both latency to emerge and foraging effort in quenda (142-146; 173-176). These results have been incorporated into the discussion (lines 197-200 and lines 223-236).*
