# Supplementary material for: Affordable RFID loggers for monitoring animal movement, activity, and behaviour
Source: PLoS One. 2022 Oct 27;17(10):e0276388. doi: 10.1371/journal.pone.0276388 (PMC9612574; doi:10.1371/journal.pone.0276388)
Supplement: Attachment — Submitted filename: comments_for_author_revised_manuscript_RFID_data_loggers_PLOS_ONE.docx [file pone.0276388.s008.docx]

The author has tried to incorporate suggestions but changes made are still not satisfactory. The manuscripts still require professional English editing. Some of the points raised earlier are still unanswered, like data for quoll study has not been correlated with camera traps or other means. Also, author is silent about failure/damage of tags ( if any ), as asked earlier. Further, in response of application of statistical technique for detailed discussion the author has applied the ANNOVA test only, no graphical presentation/ analysis is there for data collected. While the studied animals were tagged using RFID, other data about the animals (common traits and age etc.) could have been collected for detailed study. Further foraging habits/animal activity may be linked with the vegetation or any other important variable for the habitat studied.

Manuscripts still lacks the in-depth discussion, therefore, in my view the manuscripts need to be rejected.
