# Supplementary material for: Affordable RFID loggers for monitoring animal movement, activity, and behaviour
Source: PLoS One. 2022 Oct 27;17(10):e0276388. doi: 10.1371/journal.pone.0276388 (PMC9612574; doi:10.1371/journal.pone.0276388)
Supplement: Attachment — Submitted filename: ReplyToReviewers_20220902.docx [file pone.0276388.s009.docx]

We thank our two anonymous reviewers for their comments. Our overarching aim for this publication is to describe the affordable RFID readers, provide information for their construction and implementation, and to assess their performance in the field. The quoll and quenda studies are meant to act as examples of practical applications of the technology and are not the focus of the paper. For that reason, we have refrained from adding in further discussion of the specific studies and have instead focussed our discussion on the RFID technology itself, covering successes, limitations, and potential future advancements of the RFID loggers. Please see responses to each comment below.

Reviewer #1:

1. The manuscripts still require professional English editing.

*Both authors are native English speakers. We have further had two colleagues proof-read the manuscript.*

2. Some of the points raised earlier are still unanswered, like data for quoll study has not been correlated with camera traps or other means.

*We did not correlate the quoll data without camera traps or another means. This point was answered in the previous review. We responded that “For the quoll study, we did not have camera traps paired with each RFID reader, so we do not have this information”. This was also added to the manuscript in the previous revision at line 211: “Although camera trap data was not able to be collected for the quoll study to ground-truth the results, camera trap footage from the quenda study showed an 80% accuracy rate.”*

3. Also, author is silent about failure/damage of tags ( if any ), as asked earlier.

*None of the tags failed. This is detailed in the text at lines 165 and 176-177 that read “All RFID loggers functioned for 24 hours as expected” and “All RFID loggers functioned for 24 hours as expected” respectively.*

4. Further, in response of application of statistical technique for detailed discussion the author has applied the ANNOVA test only, no graphical presentation/ analysis is there for data collected.

*Thank you for the suggestion, we have added graphical representations of these results to the Supplementary Material – S5.*

5. While the studied animals were tagged using RFID, other data about the animals (common traits and age etc.) could have been collected for detailed study. Further foraging habits/animal activity may be linked with the vegetation or any other important variable for the habitat studied.

*For both studies, all animals were adults, and as they are wild, we were unable to age them in more detail than this. We were able to sex the animals and have incorporated this information into our analyses. As we were focused on trialling and validating the RFID technology, we did not collect additional data from the animals or the study site that could be incorporated into the analysis.*

6. Manuscripts still lacks the in-depth discussion, therefore, in my view the manuscripts need to be rejected.

*Our aim for this paper, is to present this economical technology, provide a guide to build it and, validate its success in the field. We feel that this discussion covers the important and interesting aspects of the technology, and that we have adequately discussed the successes, limitations, and potential future uses and advancements of the RFID loggers.*

*In the discussion, we have covered:*

- *the economic benefit of the cheaper units (including a price comparison and power analysis) at lines 187-200*
- *Limitations of the setup we employed, with suggestions on how to overcome them at lines 201-213*
- *Potential uses for this technology in ecology, including their substitution for camera traps, their use in studies of survival and dispersal, and their application in determining animal personalities (lines 214-238)*
- *Future technological advancements of such a setup, at lines 238-246.*

Reviewer #2:

1. The addressing comments are reasonably good. More technical discussion e.g. how to use the RFID data should be provided.

*Paragraphs 4-5 on the discussion (lines 214-238) are dedicated to describing uses for the data from such studies, describing their potential use in substitution for camera traps, their use in studies of survival and dispersal, and their application in determining animal personalities.*
